# Quantum-Electrodynamical Time-Dependent Density Functional Theory Description of Molecules in Optical Cavities

**DOI:** 10.1021/acs.jctc.5c01973

**Published:** 2026-02-15

**Authors:** Yetmgeta Aklilu, Matthew Shepherd, Cody L. Covington, Kalman Varga

**Affiliations:** † Department of Physics and Astronomy, 5718 Vanderbilt University, Nashville, Tennessee 37235, United States; ‡ Department of Physics and Astronomy, 606209 Auburn University, Auburn, Alabama 36830, United States; § Department of Chemistry, 2536 Austin Peay State University, Clarksville, Tennessee 37044, United States

## Abstract

We introduce a quantum-electrodynamical time-dependent density functional theory with a tensor-product representation (QED-TDDFT-TP) to model molecules strongly coupled to quantized cavity fields. By combining real-space electronic wave functions with truncated Fock-space photon states, the method captures light–matter correlations at a computational cost close to standard DFT. Benchmark calculations show good agreement with QED-FCI and QED-CASCI for ground-state energies and polaritonic spectra. Applications to weakly bound dimers—including (H_2_)_2_, Ar_2_, (H_2_O)_2_, and HF—demonstrate that cavity confinement can significantly alter binding energies and geometries in a polarization-dependent manner. The framework provides an accurate and scalable tool for studying cavity-modified molecular structure and interactions.

## Graphical abstract

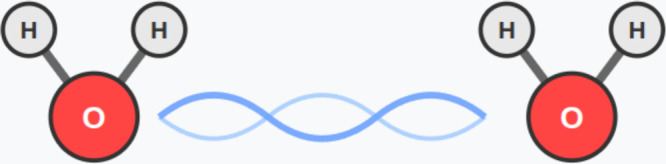

## Introduction

Cavity quantum electrodynamics (cavity QED) is a fundamental framework in quantum physics that studies the interaction between light and matter in confined electromagnetic environments. Its importance spans both theoretical understanding and practical applications. Cavity QED provides precise control over light–matter interactions by confining photons in optical cavities alongside atoms or other quantum emitters. This confinement modifies the electromagnetic environment, leading to phenomena such as the Purcell effect,^1^ where spontaneous emission rates are enhanced or suppressed depending on the cavity properties. These controlled interactions reveal fundamental aspects of quantum mechanics, including entanglement between light and matter^2–5^ and quantum superposition states.^6,7^ Light–matter coupling as a means to tune physical and chemical properties has become a major focus of experimental research.^8–21^ Theoretical investigations have also developed alongside the experimental progress.^21–64^ Several excellent review articles survey the current state of experimental and theoretical approaches to cavity light–matter interactions. These encompass reviews on hybrid light–matter states,^65–68^ *ab initio* computational methods,^33,69,70^ and molecular polaritonics.^71–73^ Describing coupled light–matter systems theoretically and computationally presents significant challenges. The quantum many-body problem involving electron–nuclear interactions is already complex, and incorporating photon degrees of freedom makes it even more demanding. Recent years have seen numerous approaches developed^44,45,47,50,51,74–85^ that extend beyond the basic two-level atom model.^86^ These methods typically build upon established many-body quantum techniques, adapting them to account for photon interactions.

The Pauli–Fierz (PF) nonrelativistic quantum electrodynamics Hamiltonian has emerged as the most practical framework^33,37,48,56,87^ for computational applications. The Pauli–Fierz Hamiltonian is the fundamental theoretical framework for describing nonrelativistic quantum electrodynamics (QED), originally developed by Wolfgang Pauli and Markus Fierz in 1938.^88–90^ The PF Hamiltonian consists of three components: the electronic Hamiltonian, the photonic Hamiltonian, and an interaction term that couples electrons and photons. The presence of this coupling term necessitates the use of a combined electron–photon wave function, where electronic states are represented through an appropriate basis set while photonic states are expressed using the Fock-space representation. The Pauli–Fierz Hamiltonian is characterized by several distinctive features. First, it operates within a nonrelativistic framework, which differs from complete relativistic QED by treating matter particles nonrelativistically while preserving the quantum nature of the electromagnetic field. Second, it employs minimal coupling^91,92^ to describe light–matter interactions, achieved by substituting the canonical momentum **p** with **p** – *e* **A**, where **A** represents the electromagnetic vector potential. Third, many practical implementations, especially in cavity QED and molecular physics, utilize the long-wavelength or dipole approximation,^93^ which considerably reduces computational complexity.

To solve the Pauli–Fierz Hamiltonian, one must include both the matter degrees of freedom and the quantized electromagnetic field. This is because the Hamiltonian contains products (couplings) of matter operators with photon-field operators, so the appropriate state space is the tensor product $H=H_{\mathrm{matter}}⊗F$ , where $H_{\mathrm{matter}}$ is the Hilbert space of the matter degrees of freedom and F is the bosonic Fock space of photons. Concretely, a state is written as a sequence of *n*-photon components Ψ = (ψ^(0)^, ψ^(1)^, ψ^(2)^, ...), where each ψ^(*n*)^ depends on the particle coordinates and spin and on *n* photon variables (momenta and polarizations), and is symmetric under exchange of the photon labels.

Similar to the Schrödinger equation, the Pauli–Fierz Hamiltonian lacks analytical solutions for multielectron atoms. For a single-electron atom or ion, the problem becomes tractable by constructing a product basis from hydrogenic eigenfunctions and Fock basis states, allowing exact diagonalization to yield the solution.^94^ However, systems with more than one electron require numerical methods for their solution.

As is typical in electronic structure calculations, methodologies can be broadly categorized into two distinct families: wave function–based methods and density–based approaches. Wave function–based methods^44,74–77,95,96^ characteristically employ coupled electron–photon wave functions, and their product structure leads to a substantial increase in computational dimensionality. The coupled electron–photon wave function can be written as

$$\left|\Psi \right\rangle =\underset{n,m}{\sum }C_{nm}\Phi_{nm}\left|n\right\rangle$$ 1

where Φ_*nm*_ is a many-body basis function representing the electrons (and nuclei, if present), |*n*⟩ is a Fock-space basis for photons, and *C*_*nm*_ is the linear expansion coefficient. The Fock-space basis can represent a single mode or multiple photon modes. The Φ_*nm*_ notation emphasizes that the spatial basis functions can be different for different photon sectors if needed.

The simplest approach, the cavity QED Hartree–Fock,^77,97^ extends the traditional Hartree–Fock method to include quantized electromagnetic field modes within optical cavities. This approach treats the coupled electron–photon system using a mean-field approximation, where electrons experience an effective field created by all other electrons and the cavity photon modes. The method employs a polaritonic wave function ansatz that is typically written as a product of an electronic Slater determinant and a photon state. refs. 42,44,76,77 employ a coupled-cluster (CC) methodology,^76,77,97^ that constructs a reference wave function from the direct product of a Hartree–Fock Slater determinant and the photon vacuum state. The ground-state QED-CC wave function is then defined by applying an exponentiated cluster operator to this product state. The primary advantage of this method lies in its systematic improvability. The traditional Complete Active Space Configuration Interaction (CASCI) approach has been also extended to include quantized electromagnetic field modes.^98^ In the CASCI ansatz for the electronic subspace, a subset of active electrons and orbitals are identified, where a full CI expansion is performed within that active space. In QED-CASCI, this framework is generalized to simultaneously treat both electronic correlation within the active space and the coupling to cavity photon modes.

The stochastic variational method (QED-SVM)^74,75,99,100^ similarly employs a product form combining matter and photonic wave functions, but differs in its treatment of the matter component through explicitly correlated Gaussian basis states. Variational parameters are optimized via stochastic selection procedures, yielding highly precise energies and wave functions. Due to the *N*! scaling of explicit antisymmetrization of the *N*-particle basis functions, the practical application of the QED-SVM approach is restricted to small atomic and molecular systems.

The density-based approach, namely quantum electrodynamical density functional theory (QED-DFT), is an extension of traditional density functional theory (DFT).^33,101–107^ QED-DFT bridges the gap between quantum optics and electronic-structure theory, making it possible to describe phenomena where light and matter interact strongly, such as in optical cavities. QED-DFT is an exact reformulation of the PF Hamiltonian, based on many-body wave theory. In QED-DFT, the complex coupled electron–photon system is represented by two uncoupled, yet nonlinear, auxiliary quantum systems. The electrons are described by the usual DFT equation which now contains potentials describing the interaction of light and matter. A separate Maxwell-like equation is used for the photons. The QED-DFT calculations mostly use real-space bases but extensions to Gaussian basis representation also exist.^108,109^ Combinations of QED-DFT with macroscopic QED,^110,111^ and extensions to Dicke^112,113^ and Rabi models^114^ have also been developed.

Standard electronic exchange-correlation (XC) functionals are inadequate for QED-DFT because they fail to account for electron-photon correlations that emerge under strong light-matter coupling. This limitation results in inaccurate predictions of polaritonic energy levels, ground-state modifications, and photon distribution statistics, making the creation of specialized QED exchange-correlation (QED-XC) potentials crucial for these systems.^101,102,105^ Recent theoretical advances—including optimized-effective-potential formulations and exact-model benchmarks—have established the formal structure of QED-XC and demonstrated the role of photon-mediated electron–electron interactions in modifying electronic structure and dynamics.^107,115–117^ These developments enable ab initio predictions of cavity-induced modifications to excitons, charge transfer, and chemical reactivity, successfully reproducing observed Rabi splittings and vacuum-field effects in molecules and materials.^82,118,119^

Our QED-DFT methodology employs a coupled electron–photon wave function analogous to those utilized in wave function–based methods. This wave function is constructed on a tensor product combining a spatial grid with a Fock-state representation. To differentiate this framework from previously discussed QED-DFT approaches, we designate the current method as QED-DFT-TP and QED-TDDFT-TP. The QED-DFT-TP represents a specific implementation of QED-DFT that adopts an alternative ansatz through the use of a coupled electron–photon wave function. While the tensor product formulation elevates the computational dimensionality, it maintains the discrete nature of quantized photon states. The coupled electron–photon wave function offers an enhanced characterization of light–matter interactions through the calculation of spatial wave functions within individual photon sectors. In this approach, each molecular orbital is paired with distinct Fock basis states representing quantized photon modes. The light–matter interaction component of the Hamiltonian governs the coupling between orbital elements across various photon states. The orthogonality of Fock states maintains the sparse structure characteristic of real-space DFT Hamiltonians. This sparsity enables the implementation of computationally efficient iterative diagonalization techniques commonly employed in conventional real-space DFT methodologies.

This paper aims to use the QED-DFT-TP methodology for computing various physical properties of molecules within optical cavities, investigate the influence of cavity parameters on these properties, and benchmark the results against established theoretical approaches. Small molecules, including LiH, BH_3_, Ar_2_, H_2_, HF and water dimers, will be used as examples.

## Formalism

The systems we consider in this paper are all nonrelativistic and as a result the light–matter coupling can be consistently described by the Pauli–Fierz nonrelativistic QED Hamiltonian.^106,120,121^ In addition, since we are working with small-sized systems, we assume that the spatial variation of the cavity field is negligible over the dimension of the system, i.e., we will use the dipole approximation. The PF Hamiltonian in the velocity gauge can be written as a sum of the kinetic energy, Kohn–Sham potential, and the photonic Hamiltonian,

$$H_{V}=\frac{1}{2m}{\left(i\hbar \nabla +eÂ\right)}^{2}+V_{\mathrm{KS}}\left(r\right)+\overset{N_{p}}{\underset{\alpha =1}{\sum }}\frac{1}{2}\left[p_{\alpha }^{2}+\omega_{\alpha }^{2}q_{\alpha }^{2}\right]$$ 2

where *V*_KS_(**r**) refers to the Kohn–Sham (KS) noninteracting potential adapted from the KS-TDDFT scheme^122^ and is given by

$$V_{\mathrm{KS}}\left(r\right)=V_{H}\left[\rho \left(r\right)\right]+V_{\mathrm{XC}}\left[\rho \left(r\right)\right]+V_{\mathrm{ion}}\left(r\right)$$ 3

where ρ is the electron density, *V*_H_ is the Hartree potential, *V*_XC_ is the exchange–correlation potential, and *V*_ion_ is the external potential due to the ions. The exchange–correlation potential *V*_XC_ is approximated using the generalized gradient approximation (GGA), developed by Perdew et al.^123^

In the long-wavelength limit, the vector potential is spatially uniform over the matter extent,

$$Â=\underset{\alpha }{\sum }A_{\alpha }\varepsilon_{\alpha }{\mathrm{q̂}}_{\alpha },A_{\alpha }\equiv \sqrt{\frac{\hbar }{\varepsilon_{0}V\omega_{\alpha }}}$$ 4

with polarization **ε**_α_, quantization volume *V*, and frequency ω_α_. The expansion of the kinetic term in ([Disp-formula eq2]) contains the paramagnetic coupling $\frac{e}{m}\mathrm{p̂}\cdot Â$ and the diamagnetic (seagull) term $\frac{e^{2}}{2m}{Â}^{2}$ . By introducing

$$\lambda_{\alpha }=\frac{\varepsilon_{\alpha }}{\sqrt{\varepsilon_{0}V}}$$ 5

the paramagnetic term becomes

$$\frac{e}{m}\underset{\alpha }{\sum }\sqrt{\frac{\hbar }{\omega_{\alpha }}}\mathrm{p̂}\cdot \lambda_{\alpha }{\mathrm{q̂}}_{\alpha }$$ 6

and the diamagnetic term

$$\frac{e^{2}}{2m}\underset{\alpha }{\sum }\frac{\hbar }{\omega_{\alpha }}\lambda_{\alpha }^{2}{\mathrm{q̂}}_{\alpha }^{2}$$ 7

The paramagnetic interaction links photon states that differ by one quantum number (Δ*n* = ±1), while the diamagnetic interaction connects photon states with quantum number changes of Δ*n* = 0, ±2. In the special case where the diamagnetic term couples photon states with identical quantum numbers (Δ*n* = 0), it behaves analogously to the dipole self-interaction (DSI) found in the length-gauge formulation, introduced below.

The Hamiltonian can also be transformed into the length gauge (see Appendix):

$$ĤL=\frac{p^{2}}{2m}+V_{\mathrm{KS}}\left(r\right)+\underset{\alpha }{\sum }\frac{\hbar \omega_{\alpha }}{2}\left({\mathrm{q̂}}_{\alpha }^{2}+{\mathrm{p̂}}_{\alpha }^{2}\right)-\underset{\alpha }{\sum }\mathrm{D̂}\cdot {Ê}_{\alpha }+\underset{\alpha }{\sum }{\left(\lambda_{\alpha }\cdot \mathrm{D̂}\right)}^{2}$$ 8

where **D** is the dipole moment and **Ê**_α_ the transverse electric field of mode α. The first term of the second line couples photon states with Δ*n* = ±1. The last term of the second line is the DSI, coupling only states with Δ*n* = 0. This term is always present and, contrary to what was believed previously, plays a crucial role in the variational formulation of the eigenvalue problem.^120^ The relation of length and the velocity-gauge Hamiltonians are further discussed in the Appendix.

The coupled system is described by orbitals defined on a tensor product of a real-space and a Fock-space. At the KS level, we can represent the orbitals as

$$\Phi_{m}=\overset{N_{F}}{\underset{n=0}{\sum }}\phi_{mn}\left(r\right)\left|n\right\rangle ,m=1,...,N_{\mathrm{occ}}$$ 9

where |*n*⟩ is the Fock-space basis for the photons, *N*_F_ is the dimension of the Fock-space, and *N*_occ_ is the number of orbitals. For this paper, we will assume that there is one dominant mode and we can ignore all others, i.e., *N*_p_ = 1. Our system is, thus, described by a four-dimensional (4D) grid: *N*_*x*_ × *N*_*y*_ × *N*_*z*_ × *N*_F_, where *N*_*x*_, *N*_*y*_, and *N*_*z*_ are the number of grid points in Cartesian real space, and *N*_F_ refers to the size of the truncated Fock-space, i.e., the vacuum state |0⟩ has *N*_F_ = 1. Because the Fock-basis states are orthogonal, the elements of the overlap matrix are given by

$$\left(\Phi_{mn}|\Phi_{m\prime n\prime }\right)=\left\langle \phi_{mn}\right|\phi_{m\prime n}\rangle \delta_{nn\prime }$$ 10

where the round bracket stands for integration over both real and Fock-space and the angle bracket is integration over only the real part,

$$\left\langle \phi_{mn}\right|\phi_{m\prime n}\rangle =\underset{ijk}{\sum }\phi_{mn}\left(x_{i},y_{j},z_{k}\right)\phi_{m\prime n}\left(x_{i},y_{j},z_{k}\right)$$ 11

The calculation of the matrix elements can be simplified further by orthogonalizing the real part of the orbitals for each Fock-state using the Gram–Schmidt method. This new orthogonal set can then be normalized

$$\overset{N_{F}}{\underset{n=0}{\sum }}|{\mathrm{\phi ̂}}_{mn}{|}^{2}=1$$ 12

where *ϕ̂*_*mn*_ with (*m* = 0, ..., *N*_occ_) represents the orthogonalized set of basis for the same photon state |*n*⟩. In the present work, the minimization for the coupled light–matter orbitals is carried out by the conjugate-gradient method. The construction of the Hamiltonian matrix in the coupled basis is described in detail in our previous work.^124^

The ground-state calculation follows conventional DFT approaches using steepest descent or conjugate gradient approaches to calculate the energies and the orbitals.

The ground state orbitals will be used to initialize the time propagation

$${\mathrm{\Phi ̂}}_{m}\left(r,t=0\right)={\mathrm{\Phi ̂}}_{m}\left(r\right)$$ 13

Any time propagation method typically used for TDDFT can be used here, and in this work we use Taylor time propagation^125^

$${\mathrm{\Phi ̂}}_{m}\left(r,t+\Delta t\right)=e^{-iH\Delta t}{\mathrm{\Phi ̂}}_{m}\left(r,t\right)=\overset{4}{\underset{j=0}{\sum }}\frac{{\left(-i\Delta t\right)}^{j}}{j!}H^{j}{\mathrm{\Phi ̂}}_{m}\left(r,t\right)$$ 14

where Δ*t* is chosen to be sufficiently small to conserve the norm of the orbitals during propagation.

To compute the absorption spectrum we apply a weak instantaneous perturbation (a “delta kick”) to the ground-state wave function,

$$\Phi_{m}^{+}=e^{i\kappa \mathrm{x̂}}\Phi_{m}$$ 15

where κ is small and *x̂* is the dipole operator. The time-dependent Schrödinger equation is then propagated, and the dipole moment *d*(*t*) = ∑_*m*_⟨Φ_*m*_(*t*)|*x̂*|Φ_*m*_(*t*)⟩ is recorded. The absorption spectrum is obtained from the imaginary part of the Fourier transform of the dipole response,

S(ω)∝ωIm[∫0Teiωtd(t)dt] 16

## Results

The primary objective of these calculations is to benchmark the QED-DFT-TP approach against other established methods across various molecules and molecular dimers. This comparison is particularly timely given the rapid development of numerous new approaches in this field. To ensure a correct comparison, we have employed identical parameters across all methods, including coupling strengths, cavity frequencies, and molecular geometries. We represent **λ** = λ**ε**, where **ε** is a unit vector describing the polarization of the cavity mode, e.g., (1, 0, 0), and λ is the coupling strength. In this work, we will use λ ≤ 0.1, which, according to $\lambda =1/\sqrt{\varepsilon_{0}V_{\mathrm{eff}}}$ , corresponds to subnm^3^ effective volumes.^126,127^ This range coincides with volumes achieved in picocavity experiments.^126,127^ The velocity-gauge Hamiltonian is employed in all calculations. The velocity gauge allows more straightforward analysis of photonic Fock state populations compared to most ab initio cavity QED approaches. The latter typically use the length gauge and therefore require Power–Zienau–Woolley transformation of the ladder operators to extract physical photonic Fock state populations. The differences of results in velocity and lengths gauges are discussed in Appendix A. Since this calculation employs a finite-difference grid to represent the wave functions, the computed total energies are sensitive to the alignment between grid points and ionic positions. To maintain consistency when investigating energy as a function of intermolecular distance, we preserve the relative positioning by ensuring that molecular coordinates remain commensurate with the underlying computational lattice. This constraint limits our ability to position molecules at arbitrary locations; instead, molecular displacements are restricted to shifts with integer multiples of the grid spacing.

### Selected Test Cases

This work examines the following 6 molecular systems under cavity quantum electrodynamics conditions. We begin by revisiting the potential energy surface (PES) of LiH, which was previously explored in our earlier study.^124^ Recent developments^128^ now provide additional benchmarks for comparison with our methodology. The second system of interest is BH_3_ for which we calculate the Rabi splitting via time propagation of molecular orbitals to obtain the absorption spectrum, enabling comparison with the recent work of ref 128. Subsequently, we investigate the PES of an H_2_ dimer to characterize cavity-induced modifications to intermolecular forces, with results benchmarked against those reported in ref 97. A parallel study of the Ar dimer allows us to compare our approach with QED-DFT calculations from ref 117, providing a valuable cross-validation between distinct QED-DFT formulations. We then examine cavity effects on hydrogen bonding by computing the PES of a water dimer, comparing our findings with ref 128. Finally, we explore the orientational dependence of the cavity-modified PES for an HF dimer, a system not previously characterized in the literature. The molecular geometries and other parameters of the calculation are listed in the Supporting Information for each system.

### LiH

In this section, we model the ground-state potential energy surface of LiH and compare our results with Photon-Number Quantum Electrodynamical Full Configuration Interaction (PN-QED-FCI) calculations from ref 128. The LiH molecule is oriented with its internuclear axis parallel to the polarization vector of a cavity mode with frequency ω = 0.121 au This frequency is chosen to be resonant with the molecule’s lowest singlet excitation.^128^

Figure 1 presents a comparison of PN-QED-FCI, and QED-DFT-TP results. The QED-DFT-TP calculation fully converges using *N*_F_ = 4. Only the lowest Fock states exhibit significant coupling to the electronic subsystem, with the |0⟩ state accounting for approximately 98% of the total photonic probability. To facilitate visual comparison, the QED-DFT-TP energies have been uniformly shifted by Δ = −7.264 hartree. All computational parameters were selected to match those employed in the PN-QED-FCI benchmarks of ref 128. The agreement between methodologies is excellent, with nearly identical predictions for the equilibrium bond length. The primary discrepancy emerges in the dissociation limit, which may stem from either the compact 6-311G basis set employed in the wave function methods or limitations of the GGA exchange-correlation functional in QED-DFT-TP.

**Figure 1:**
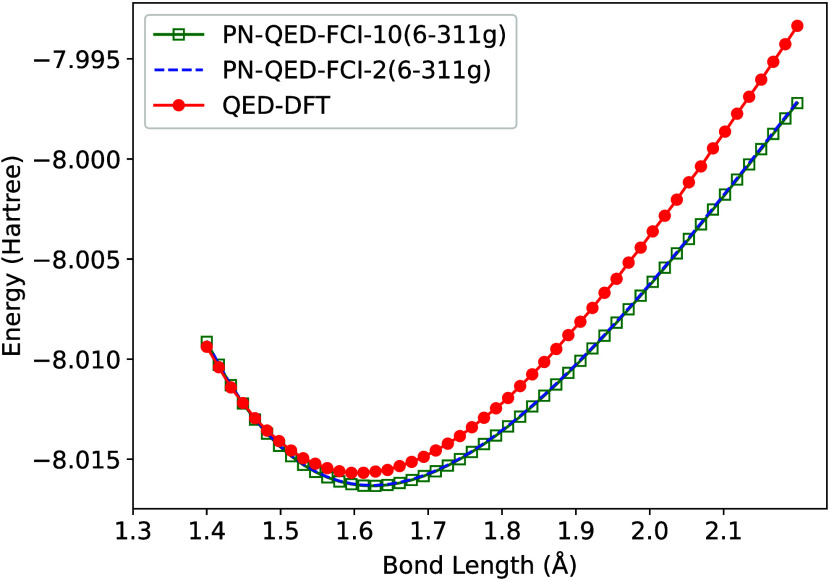
Comparison of LiH PES calculated using QED-DFT-TP with PN-QED-FCI. All graphs use λ = 0.05 and ω = 0.121. The PN-QED-FCI-*N*_F_ calculation used *N*_F_ = 2 and *N*_F_ = 10, and the QED-DFT-TP results were calculated using *N*_F_ = 4.

We quantified the agreement between potential energy surfaces obtained with different light–matter electronic-structure methods using the nonparallelity error (NPE), defined as the difference between the maximum and minimum absolute energy deviations along a given reaction coordinate. This metric isolates differences in shape between two PESs, independent of constant vertical energy offsets, and therefore provides a stringent test of whether two approaches predict the same underlying physics across configuration space. For the LiH case, the calculated NPE between the PN-QED-FCI and QED-DFT-TP is approximately 0.3 eV, indicating that the curves are nearly parallel over the full bond-length range. The residual nonparallelity arises from a small curvature mismatch rather than from systematic distortions of the PES.

Figure 2 presents the ground-state energy of LiH as a function of bond length for various coupling strengths λ and cavity frequencies ω. The molecular system is coupled to both the |0⟩ and |1⟩ photon Fock states.

**Figure 2:**
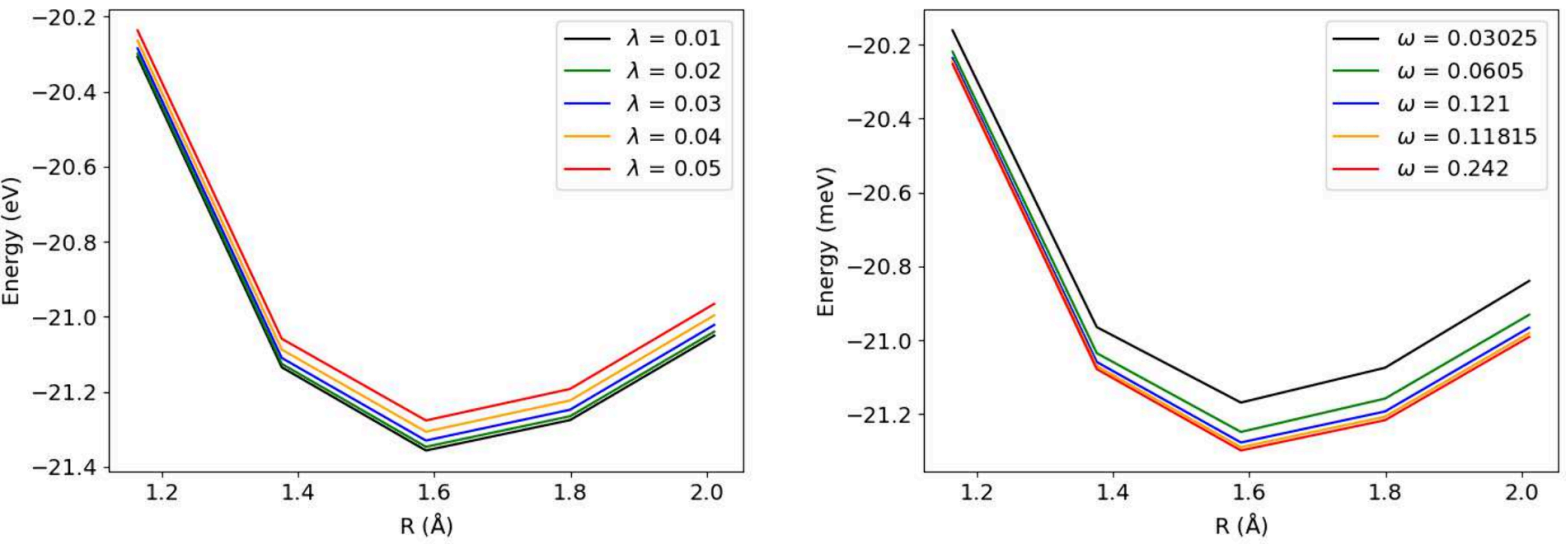
LiH: Ground-state energy vs bond length for ω = 0.121 for λ = 0.01, 0.02, 0.03, 0.04, and 0.05 (left). Ground-state energy vs bond length for ω = 0.03025, 0.0605, 0.121, 0.1815, and 0.242 while keeping λ = 0.05 (right).

The results demonstrate that increasing λ elevates the ground-state energy due to the diamagnetic contribution in the Pauli–Fierz Hamiltonian. Notably, the overall shape of the potential energy curves remains qualitatively unchanged across different coupling strengths, exhibiting primarily a vertical shift that scales approximately as 2λ^2^. This behavior indicates that the diamagnetic self-energy contributes a nearly constant offset across all bond lengths.

In contrast, increasing ω reduces the ground-state energy (Figure 2), producing an opposite effect on the system’s energetics. This inverse relationship arises because the diamagnetic term, which dominates the energy modification, exhibits an inverse dependence on the cavity frequency. Figure 3 illustrates the variation in |1⟩ state occupation, which quantifies the photonic excitation from the |0⟩ state. The bond-length dependence of the |1⟩ occupation follows a trend similar to that observed for the energy in Figure 2. The occupation increases substantially with larger λ values; note that the occupations in Figure 3 are scaled by different multiplicative factors to facilitate visual comparison on a single plot. The cavity frequency dependence of the |1⟩ occupation (Figure 3) exhibits behavior similar to the energy variation for ω = 0.121 au and ω = 0.1815 au However, for larger ω values, the occupation decreases with increasing internuclear separation. This is likely due to the fact that increasing the frequency ω raises the energy of the nn nth photonic mode according to ℏω. This increased energy separation between states diminishes the effective coupling strength between the |0⟩ state and higher photon number states |*n*⟩, consequently reducing the occupation probability of these higher-lying states.

**Figure 3:**
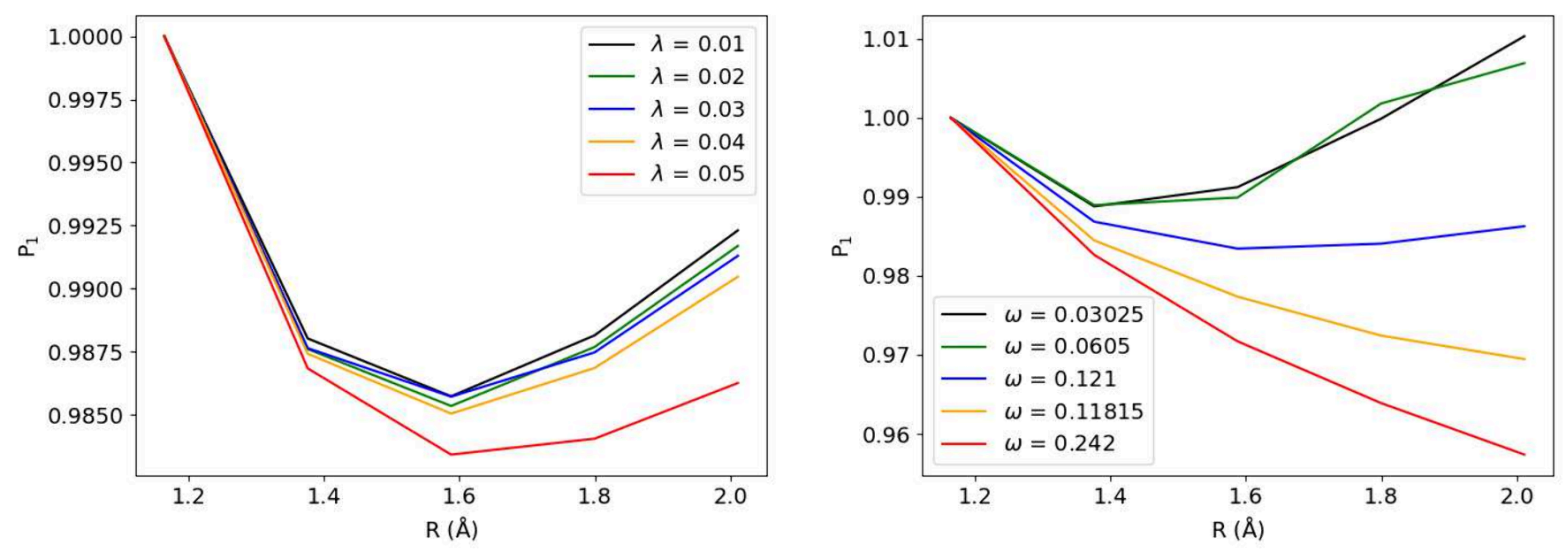
Dependence of the occupation of photon space |1⟩ on λ and ω for different bond lengths.

### BH_3_

In this section, we compare our calculated Rabi splitting for BH_3_ with QED-FCI results from ref 128. While the QED-FCI approach determines the Rabi splitting by directly diagonalizing the Hamiltonian to obtain the energy separation between upper and lower polariton states, our methodology employs an alternative strategy. We compute the absorption spectrum via real-time propagation and extract the Rabi splitting from the spectral positions of the polaritonic peaks.

Our TDDFT time-propagation approach evaluates the absorption spectrum by applying a brief delta-function electric field pulse to the ground-state system, then propagating the time-dependent Kohn–Sham equations to track the subsequent evolution of the electronic density. The time-dependent induced dipole moment is recorded throughout the propagation, from which we obtain the frequency-dependent polarizability through Fourier transformation. The absorption spectrum is then derived from the imaginary component of the polarizability, which corresponds directly to the optical absorption cross-section. The Rabi splitting is identified as the frequency separation between the upper and lower polariton peaks in this spectrum.

The cavity-free case exhibits an excitation peak at 0.4732 au (see Figure 4), which closely matches the molecule’s third singlet excited state reported by Vu et al. (2024).^128^ Initially, we couple the molecule to the |0⟩ Fock-state. Although this configuration lacks light–matter coupling, the excitation peak energy increases due to the positive diamagnetic term, as demonstrated in Figure 4. To determine the upper and lower peak positions, we set the cavity frequency ω equal to *E*/*ℏ*, where *E* represents the absorption peak energy (middle curve in Figure 4). Since the diamagnetic term causes the peak position to shift with varying λ, we correspondingly adjust ω in our calculations. When light–matter coupling is introduced, the single absorption peak splits into two distinct peaks. The energies of these split peaks define the upper and lower polaritons.

**Figure 4:**
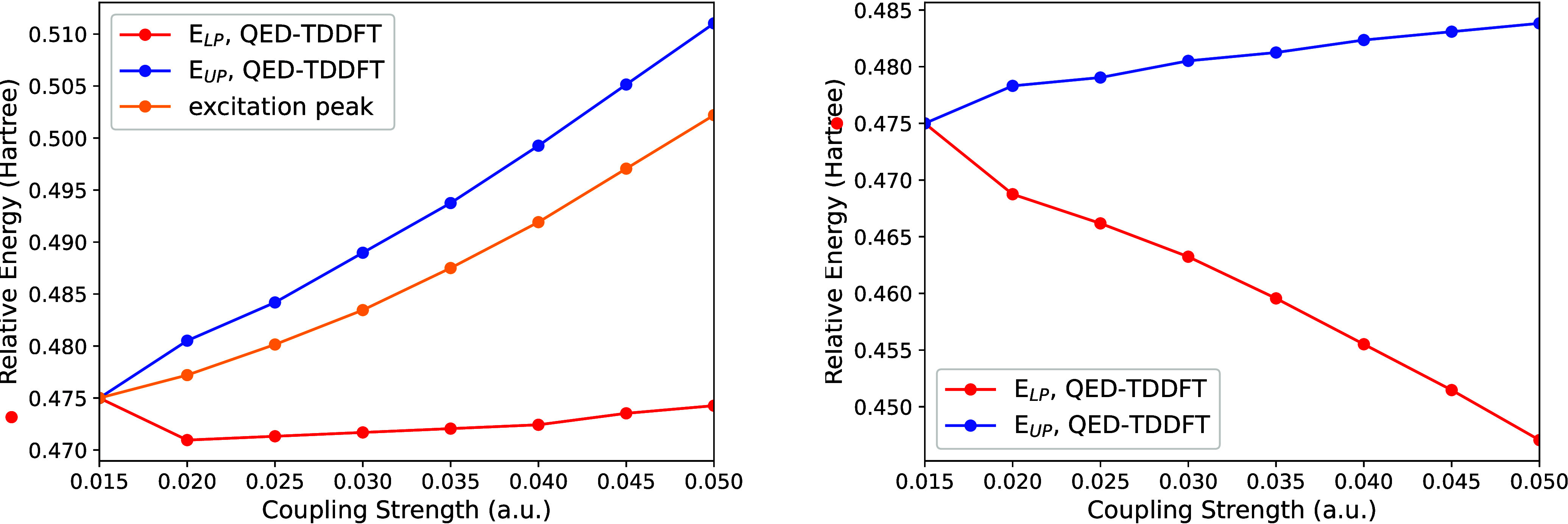
Left: Dependence of the polaritonic energies on λ. The upper and lower curves show the energies of the upper and lower polaritons; the middle curve shows the energy dependence due to the diamagnetic term without coupling to the light. Right: Upper and lower polaritonic energies after subtracting the diamagnetic term’s effect.

Figure 4 illustrates the λ dependence beginning at λ = 0.015. Below this threshold value, peak splitting is not observed. This limitation arises because QED-TDDFT-TP produces absorption spectra with peaks of finite width, causing overlap at small λ values. While extending the propagation time could potentially resolve this issue, such calculations would be computationally impractical. As anticipated, the upper polariton energy increases with λ. However, the lower polariton energy exhibits minimal variation. This behavior occurs because the peak frequencies shift upward with increasing λ, creating the appearance that *E*_LP_ remains constant. To provide a clearer visualization, the right panel of Figure 4 shows the polariton energies after removing the diamagnetic contribution (by subtracting the middle curve from both the upper and lower curves). With this adjustment, both upper and lower polaritonic energies display the expected λ-dependent behavior.

Figure 5 presents a comparison of polaritonic energies for BH_3_ calculated using QED-TDDFT and QED-FCI methods for the same excited state. The QED-FCI results are taken from Vu et al. (2024).^128^ The graph reveals that while both methods demonstrate increasing relative energy with rising λ values, QED-TDDFT-TP consistently underestimates the splitting magnitude and exhibits greater nonlinear behavior compared to QED-FCI. Additionally, our approach underestimates the upper polariton energy, but the overall agreement between the QED-FCI and QED-TDDFT-TP is encouraging.

**Figure 5:**
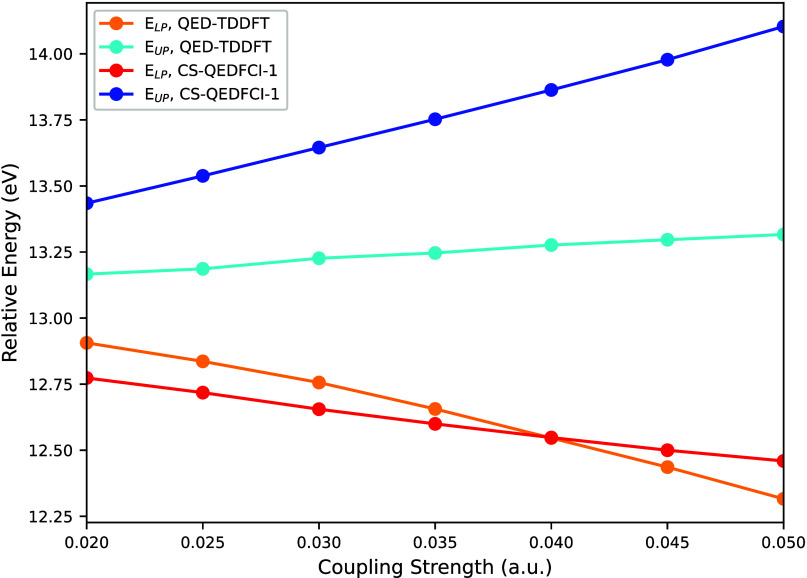
Comparison of the polaritonic energies of BH_3_ using QED-TDDFT and QED-FCI. The QED-TDDFT graph was shifted upward by 0.15 eV to fit it into the same scale. The graph begins at λ = 0.02 because for lower coupling strengths we do not resolve a splitting in QED-TDDFT.

### (H_2_)_2_

Intermolecular forces can be fundamentally understood as electronic interactions mediated by transverse electromagnetic fields.^97,129^ Consequently, modifying electromagnetic boundary conditions through cavity confinement can substantially alter intermolecular interactions.^130^ Moreover, in the strong coupling regime, molecules can interact with one another via delocalized cavity photons even at separations where direct Coulombic interactions become negligibly weak. Figure 6 presents the potential energy surface of (H_2_)_2_ computed using QED-DFT-TP, compared with QED-FCI results from ref 97. The QED-DFT-TP method reproduces the overall topology of the QED-FCI surface and correctly predicts that cavity modes with ϵ_*z*_ polarization (parallel to the intermolecular axis) yield lower binding energies than those with ϵ_*x*_ polarization (perpendicular). However, QED-DFT-TP systematically overestimates the binding energy across all configurations. The comparison between QED-DFT and QED-FCI reveals substantially larger shape discrepancies. For x-polarized coupling the NPE is approximately 6 meV, while for z-polarization it increases slightly to about 6.5–7 meV. In both polarizations, the two methods coincide in the dissociation limit but differ markedly near the equilibrium region, where QED-DFT predicts a significantly deeper minimum than QED-FCI. Consequently, the NPE is dominated by the depth mismatch at short and intermediate separations, indicating that the PESs are not parallel. This behavior reflects an overbinding tendency of QED-DFT relative to the wave function reference in the cavity-modified van der Waals regime. For deeper understanding of the differences convergence studies of the FCI approach would be valuable.

**Figure 6:**
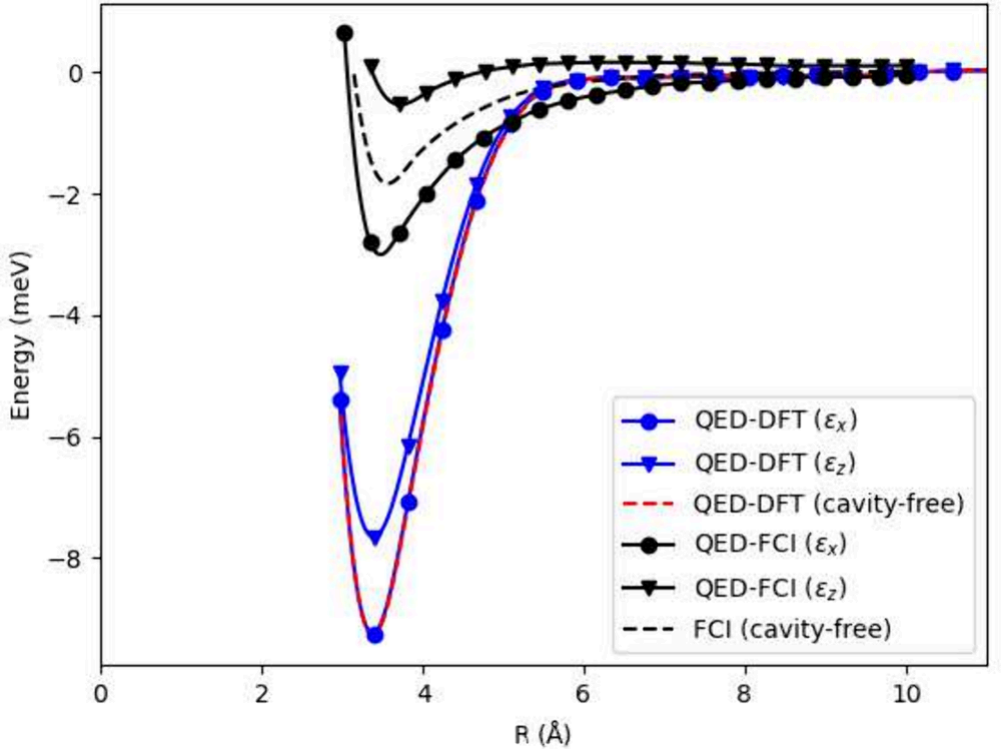
Potential energy surface for (H_2_)_2_: λ = 0.05, ω = 12.7 eV. Each H_2_ molecule is aligned along the *x*-axis with a H–H distance of 1.4 au, centered at *z* = ±*R*/2.

A notable discrepancy is that QED-DFT-TP predicts the ϵ_*x*_ polarization to produce a potential energy surface nearly identical to the cavity-free case, whereas QED-FCI shows substantial cavity modification for this polarization. It is important to recognize that in the cavity-free limit, our method reduces to conventional DFT, which cannot accurately describe van der Waals interactions despite yielding qualitatively reasonable curves.^131^ We also note that the QED-DFT approach employed in ref 97 does not appear to reproduce a bound potential energy minimum for this system.

Several factors may account for the stronger binding predicted by QED-DFT-TP relative to QED-FCI. First, there is a difference in reference energies: QED-DFT-TP employs a reference state with the two water clusters separated by 200 Å, whereas such large separations are computationally intractable for QED-FCI, necessitating a 30 Å reference separation instead. Additional discrepancies could arise from the approximate exchange-correlation functional used in the DFT approach or from basis set truncation effects in the FCI calculation.

### Ar_2_

We have also investigated cavity-modified intermolecular interactions in Ar_2_. The two argon atoms are positioned along the *z*-axis with varying separation distance *R*. In the cavity-free case (λ = 0), our results closely match those of ref 117, predicting an equilibrium separation of 3.9 Å, although our binding energy is approximately 5 meV stronger. As shown in Figure 7, the binding energy remains nearly unchanged when the cavity polarization **ϵ** is perpendicular to the dimer axis and the coupling strength is weak (λ = 0.05). This behavior can be understood by examining the occupation probability of the |1⟩ Fock state (Figure 7), which exhibits minimal variation with internuclear separation, leaving the intermolecular potential essentially unmodified relative to the cavity-free case. Increasing the coupling strength to λ = 0.1 introduces a stronger distance dependence in the |1⟩ state occupation (Figure 7), resulting in reduced binding energy compared to the cavity-free reference.When the cavity polarization is parallel to the dimer axis, the occupation of the |1⟩ state exhibits pronounced sensitivity to the interatomic separation, and the binding energy decreases substantially with increasing λ.

**Figure 7:**
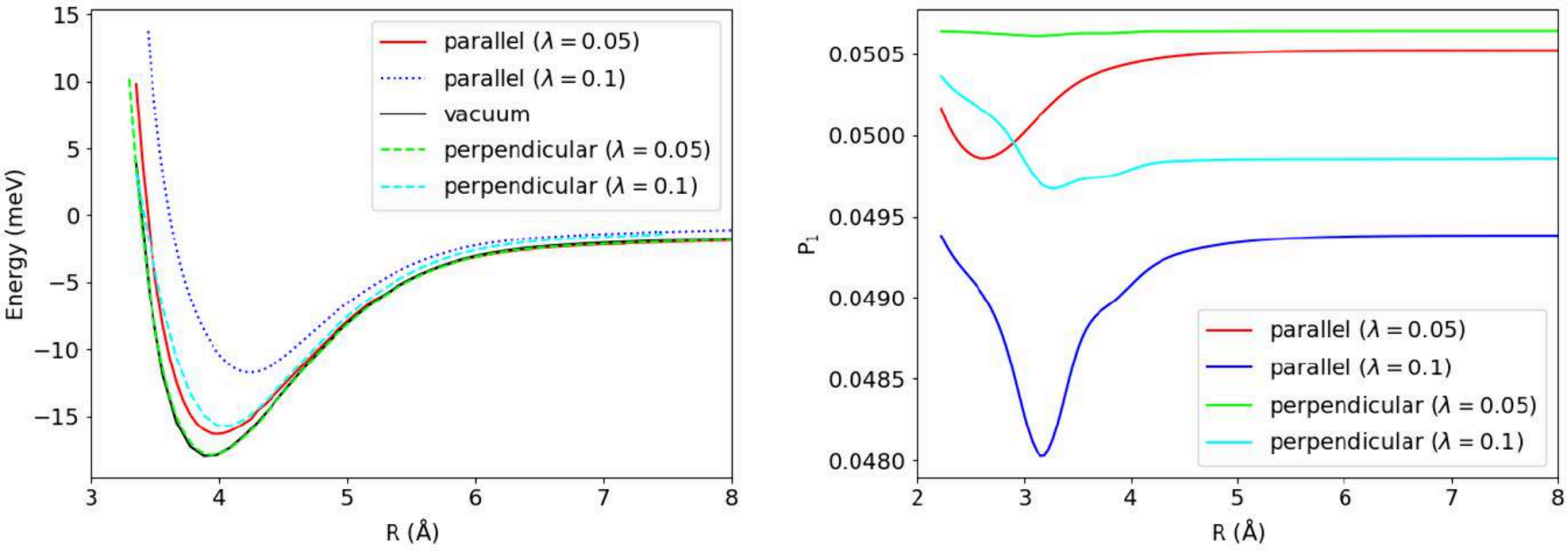
Potential energy surface (left) and occupation probability of the |1⟩ Fock-space (right) for Ar_2_. To enable comparison within a single figure, the occupation probabilities corresponding to λ = 0.05 have been offset by adding 0.38. ω = 0.467 au was used in the calculations.

Figure 8 displays the Ar dimer energy as a function of interatomic distance using parameters λ = 0.1 and ω = 0.0375 atomic units, identical to those employed in ref 117. When compared to Figure 7, the perpendicular configuration exhibits enhanced binding energy that approaches the cavity-free binding energy more closely, which results from the 10-fold reduction in frequency. Conversely, the parallel configuration shows reduced binding relative to the previous case. ref 117. predicted that parallel configurations would be less bound than the cavity-free case, while perpendicular configurations would be more strongly bound than the cavity-free case. Our findings are consistent with ref 117. regarding the parallel configuration but disagree concerning the perpendicular configuration. This difference can be attributed to several methodological distinctions. While we employ GGA functionals, the alternative QED-DFT approach utilizes hybrid functionals. Additionally, our method incorporates photonic effects through a tensor product representation, whereas the other approach implements photon many-body dispersion within the Hamiltonian.

**Figure 8:**
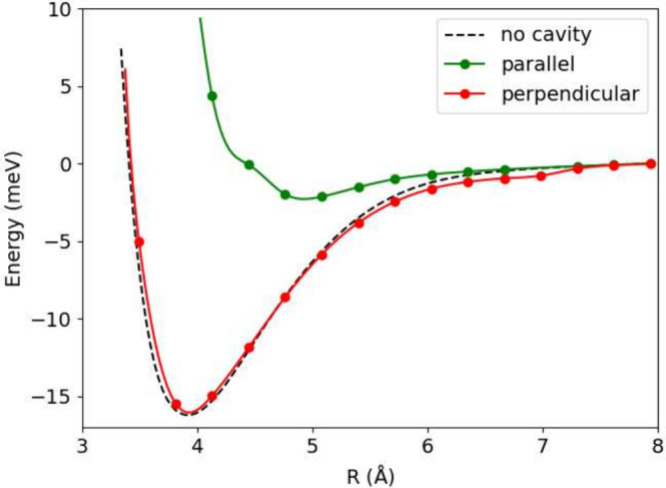
Potential energy surface for Ar_2_. λ = 0.1 and ω = 0.0375 au used in the calculations.

### (H_2_O)_2_

We investigated the influence of cavity confinement on hydrogen bonding in a water dimer by varying the oxygen–oxygen separation distance RR R. This system was recently examined in ref 97. Since the exact atomic coordinates from that study were not available, we constructed two geometrically similar configurations based on the structures depicted in ref 97, as shown in Figure 9. The cavity polarization is oriented along the O–O axis, and the cavity frequency and coupling strength are set to match those of ref 97: ω = 7.86 eV and λ = 0.1.

**Figure 9:**
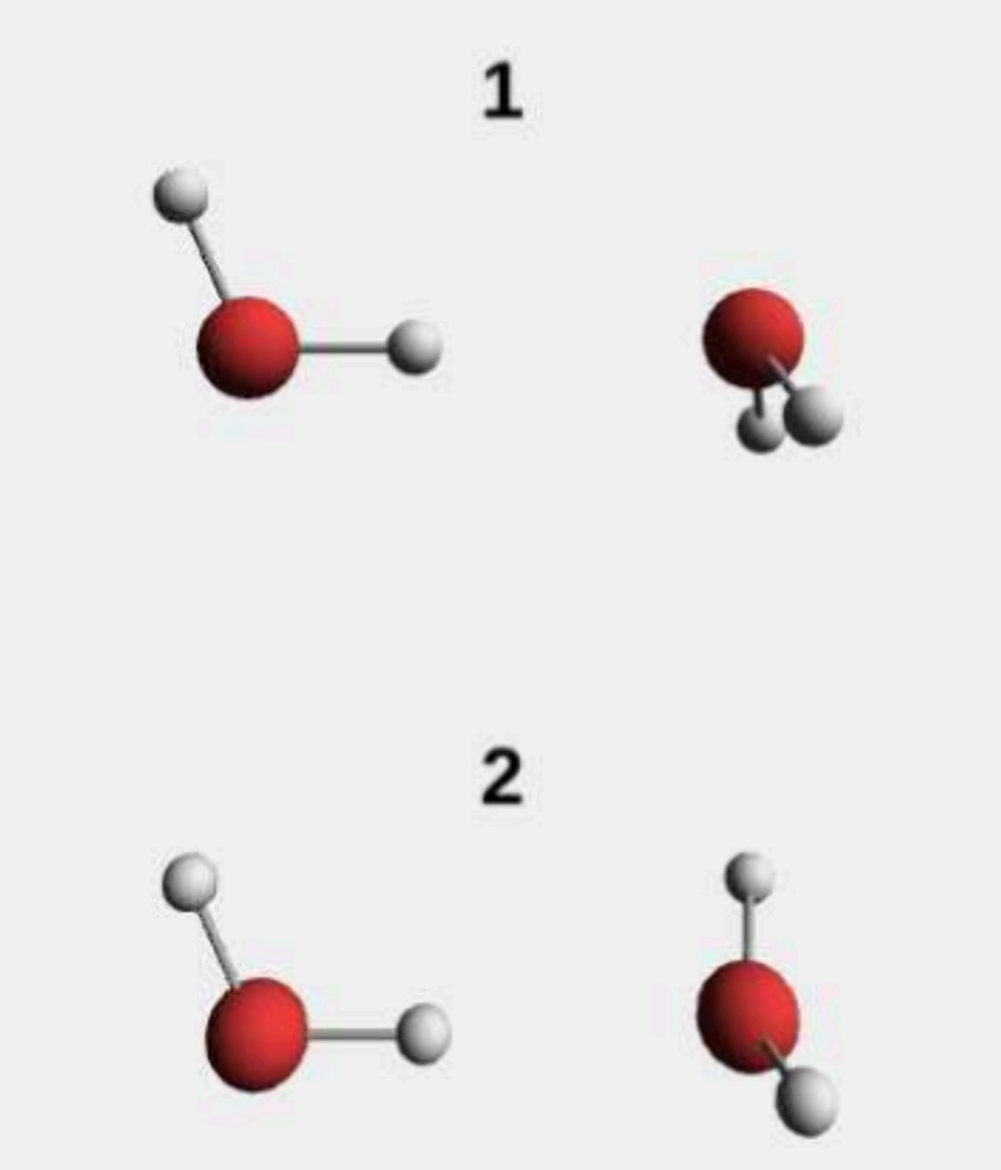
Two water dimer configurations used in the calculations.

Figure 10 shows that our potential energy curve exhibits excellent agreement with the Coupled-Cluster Singles and Doubles (CCSD) results from ref 97 in the cavity-free case. Both CCSD and QED-DFT-TP predict an equilibrium separation of 2.9 Å. The binding energies are also in good agreement: CCSD yields 214 meV (Figure 6 in ref 97), while QED-DFT-TP predicts 240 meV. Under cavity confinement, CCSD calculations indicate a modest 30 meV reduction in binding energy, whereas QED-DFT-TP predicts a substantially larger decrease of approximately 130 meV. This more pronounced modification is physically reasonable given the strong light–matter coupling regime accessed by the chosen parameters. The quantitative discrepancy between the two approaches likely stems from differences in the water dimer geometries employed. This interpretation is supported by the substantial variation in cavity-induced binding energy changes observed between our two distinct water dimer configurations, which underscores the geometric sensitivity of cavity effects on hydrogen bonding.

**Figure 10:**
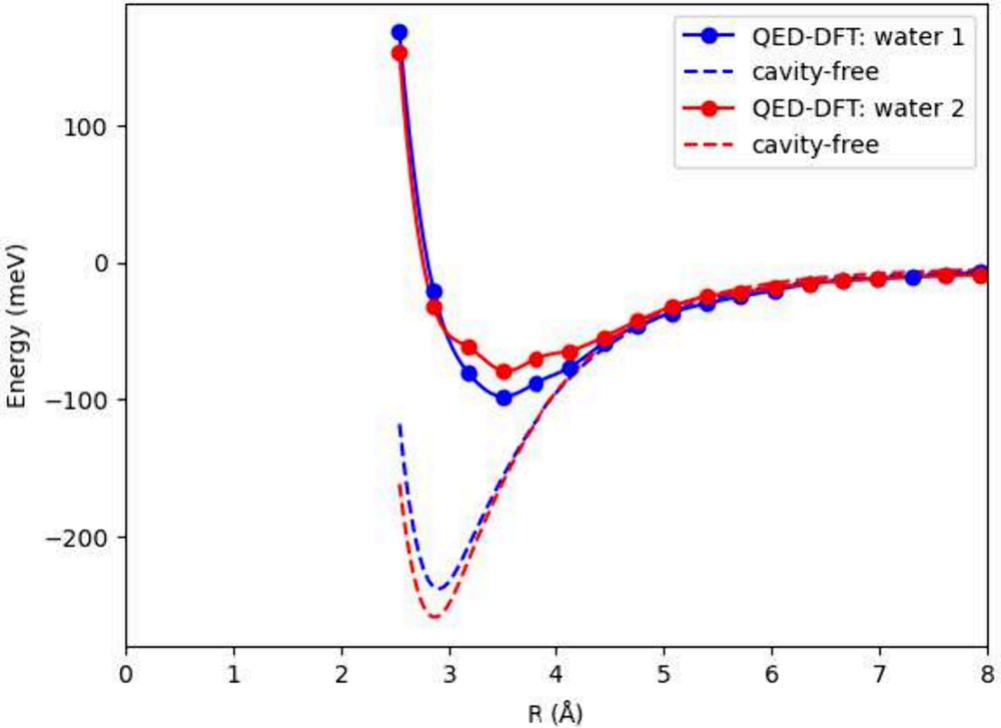
Potential energy surfaces for (H_2_O)_2_ systems.

### HF Dimer

We examined the distance-dependent binding energy of HF dimers in two distinct molecular arrangements: parallel orientation (HF–HF) and antiparallel orientation (HF–FH), as illustrated in Figure 11. The HF molecular axes are oriented along the *x*-direction. Two cavity polarization configurations were investigated: **ϵ** = (1, 0, 0), designated as “perpendicular” since it is orthogonal to the intermolecular *z*-axis, and **ϵ** = (0, 0, 1), termed “parallel” as it aligns with the axis connecting the two molecules.

**Figure 11:**
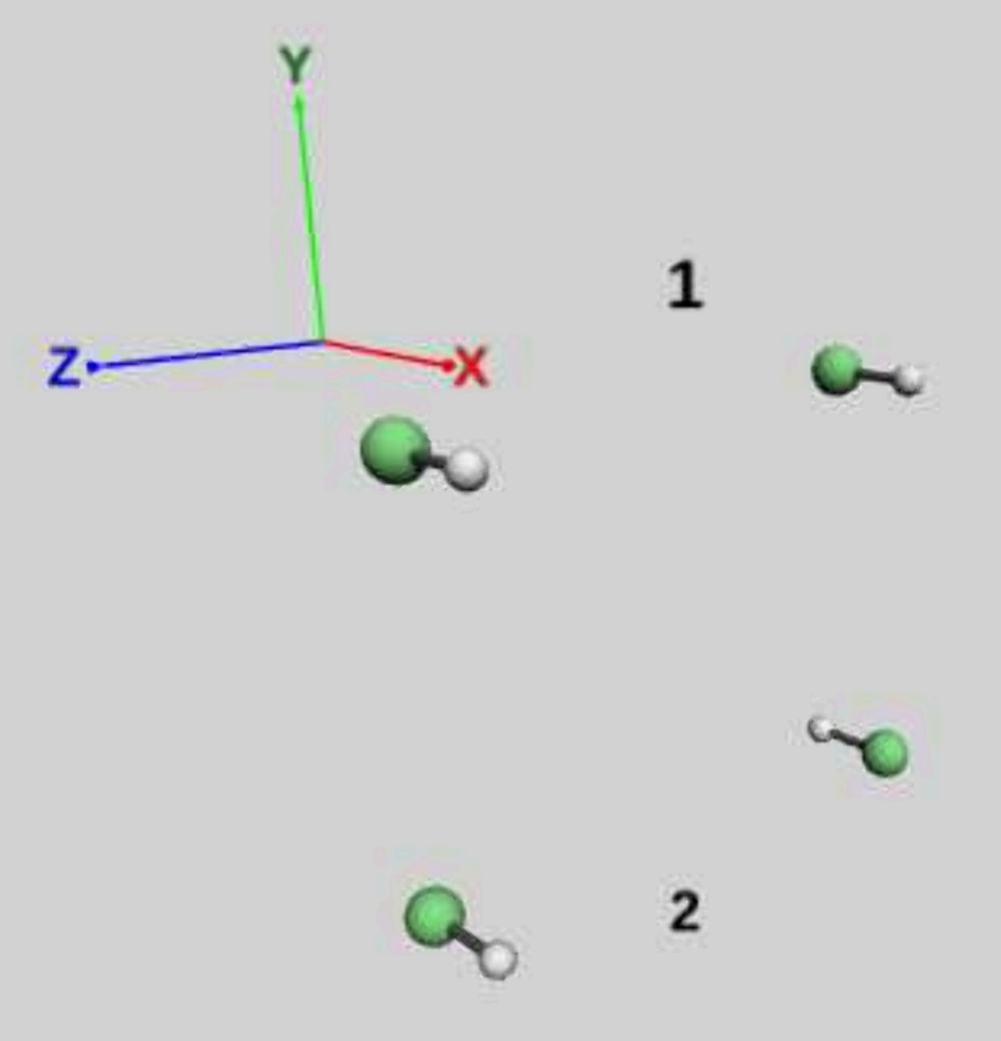
Two HF dimer configurations used in the calculations.

For the parallel HF–HF configuration, Figure 12 demonstrates that the cavity-free system exhibits no intermolecular binding, and light–matter coupling in either polarization fails to induce molecular association. The parallel polarization yields higher total energies than the perpendicular case, while both polarizations produce minimal |1⟩ photon-state occupation across all intermolecular separations.

**Figure 12:**
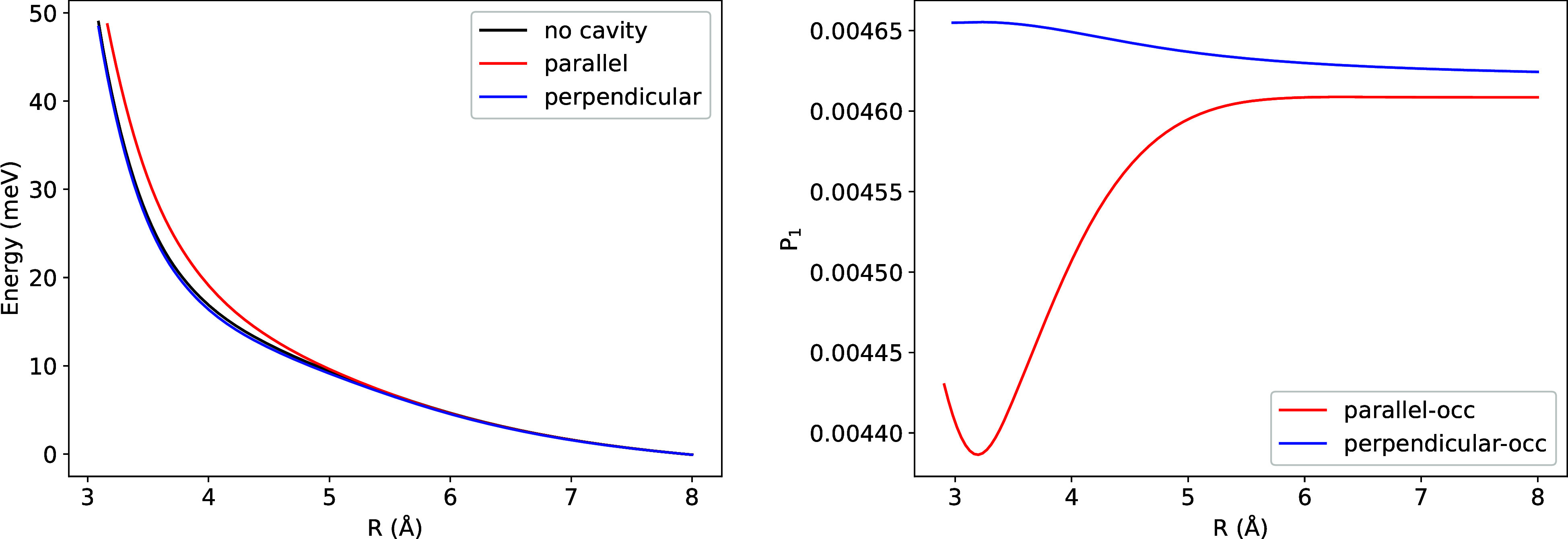
Potential energy surface (left) and occupation probability of the |1⟩ Fock-space (right) for the HF dimer (dimer 1 in Figure 11).

In contrast, the antiparallel HF–FH arrangement (Figure 13) exhibits substantial intermolecular binding with an energy minimum at approximately 2.7Å—notably about three times the HF monomer bond length of 0.92 Å. Light–matter coupling reduces the binding strength for both polarization directions relative to the cavity-free case. Parallel polarization decreases the equilibrium separation, while perpendicular polarization increases it. The |1⟩ photon-state occupation in the antiparallel configuration is significantly enhanced compared to the parallel arrangement. The occupation profiles exhibit polarization-dependent behaviors: at short intermolecular distances they differ substantially but converge at large separations. Notably, the parallel polarization exhibits an occupation minimum near 3 Å, whereas the perpendicular polarization shows monotonic behavior with no such feature across the investigated distance range.

**Figure 13:**
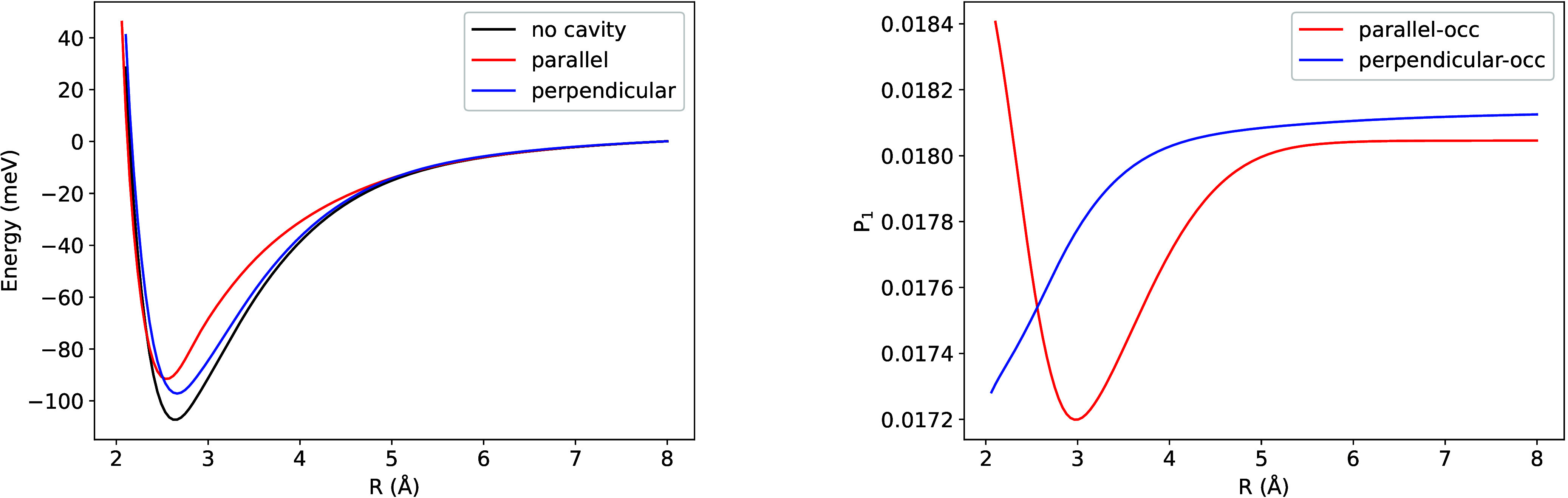
Potential energy surface (left) and occupation probability of the |1⟩ Fock-space (right) for the HF dimer (dimer 2 in Figure 11).

## Summary

This work examines the effectiveness of combining quantum electrodynamics with time-dependent density functional theory to model molecular systems under strong coupling conditions within optical cavities, benchmarking the results against existing theoretical approaches. Conventional density functional theory is extended by incorporating the Pauli–Fierz nonrelativistic quantum electrodynamics Hamiltonian, with the coupled electron–photon system represented on a tensor product of real-space and Fock-space. The method is benchmarked against established wave function approaches including photon-number quantum electrodynamical full configuration interaction and complete active space configuration interaction for small molecular systems. Potential energy surfaces for LiH are shown to demonstrate excellent agreement with reference calculations, while Rabi splitting calculations for BH_3_ exhibit qualitative agreement despite some quantitative differences. The influence of cavity parameters on intermolecular interactions is investigated through studies of hydrogen-bonded and van der Waals dimers, including (H_2_)_2_, Ar_2_, (H_2_O)_2_, and (HF)_2_ systems. Cavity coupling is found to significantly modify binding energies, equilibrium distances, and photon occupation numbers, with strong dependence on molecular orientation relative to field polarization observed. For HF dimers, parallel (HF–HF) configurations are shown to remain unbound in cavities while antiparallel (HF–FH) arrangements exhibit substantial binding that decreases upon light–matter coupling.

The QED-TDDFT-TP approach demonstrates computational tractability while maintaining sufficient accuracy to describe cavity QED effects and polariton physics, reproducing the qualitative features of strong coupling regimes. Residual quantitative discrepancies relative to wave function calculations warrant further analysis of finite photon-basis effects and density-functional approximation quality.

The QED-DFT-TP method presented in the paper is orders of magnitude cheaper than both QED-CC and especially QED-FCI. It retains explicit quantized photon states while keeping computational cost close to standard DFT, enabling simulations of realistic molecules and dimers under strong light–matter coupling.

The present work assumes a bare matter exchange correlation functional which is the same in each Fock-space. This approximation could be a source of error relative to the exact solution. Future work is needed to develop suitable exchange-correlation functionals for the coupled electron orbitals Fock space approach.

## Supplementary Material



## Data Availability

The data that support the findings of this study are available from the corresponding author upon reasonable request.

## Appendix

### Length and Velocity Gauge

The goal of this appendix is to give a brief overview of the different forms of the Pauli–Fierz Hamiltonian and gauge invariance.^28,51,120^ Consider matter (atoms or molecules) interacting with quantized transverse radiation in the Coulomb gauge. In the nonrelativistic limit and long-wavelength (dipole) approximation, the velocity-gauge Pauli–Fierz Hamiltonian reads

$${Ĥ}_{V}=\overset{N_{e}}{\underset{i=1}{\sum }}\frac{1}{2m_{i}}{\left({\mathrm{p̂}}_{i}-q_{i}Â\right)}^{2}+{\mathrm{V̂}}_{\mathrm{Coul+ext}}+\underset{\alpha }{\sum }\frac{\hbar \omega_{\alpha }}{2}\left({\mathrm{p̂}}_{\alpha }^{2}+{\mathrm{q̂}}_{\alpha }^{2}\right)$$
17

*V̂̂*_Coul+ext_ collects Coulomb interactions and external potentials. Each photonic mode α is a harmonic oscillator with canonical pair (*q̂*_α_, *p̂*_α_), obeying [*q̂*_α_, *p̂*_β_] = *i*δ_αβ_. In the long-wavelength limit, the vector potential is spatially uniform over the matter extent,

$$Â=\underset{\alpha }{\sum }A_{\alpha }\varepsilon_{\alpha }{\mathrm{q̂}}_{\alpha },A_{\alpha }\equiv \sqrt{\frac{\hbar }{\varepsilon_{0}V\omega_{\alpha }}}$$
18

with polarization **ε**_α_, quantization volume *V*, and frequency ω_α_. Define the total matter dipole operator without charges,

$$\mathrm{R̂}\equiv \overset{N_{e}}{\underset{i=1}{\sum }}r_{i}-\overset{N_{n}}{\underset{j=1}{\sum }}Z_{j}R_{j}$$
19

so that the physical dipole is **D̂** = ∑_*i*_ *q*_*i*_ **r**_*i*_ = −*e*∑_*i*_ **r**_*i*_ + ∑_*j*_ *Z*_*j*_ *e* **R**_*j*_. For brevity, many steps below are shown for a single electron with charge −*e*; the generalization is straightforward. First we introduce the Power–Zienau–Woolley (PZW) transformation:^132,133^

$$Û\equiv \exp\left[i\underset{\alpha }{\sum }g_{\alpha }\left(\varepsilon_{\alpha }\cdot \mathrm{R̂}\right){\mathrm{q̂}}_{\alpha }\right],g_{\alpha }\equiv \frac{e}{\sqrt{\varepsilon_{0}V\hbar \omega_{\alpha }}}$$
20

It effects simple shifts on the canonical operators:

$${Û}^{†}{\mathrm{q̂}}_{\alpha }Û={\mathrm{q̂}}_{\alpha }$$
21

$${Û}^{†}{\mathrm{p̂}}_{\alpha }Û={\mathrm{p̂}}_{\alpha }+g_{\alpha }\varepsilon_{\alpha }\cdot \mathrm{R̂}$$
22

$${Û}^{†}{\mathrm{p̂}}_{i}Û={\mathrm{p̂}}_{i}+eÂ$$
23

$${Û}^{†}ÂÛ=Â$$
24

where we used $Â=\underset{\alpha }{\sum }A_{\alpha }\varepsilon_{\alpha }{\mathrm{q̂}}_{\alpha }$ and $eA_{\alpha }=\hbar g_{\alpha }$ so that *e* **Â** = *ℏ* ∑_α_ *g*_α_ ***ε***_α_ *q̂*_α_. Using eqs 22–24, one finds

$${Û}^{†}\left({\mathrm{p̂}}_{i}-\left(-e\right)Â\right)Û={\mathrm{p̂}}_{i}$$
25

$${Û}^{†}\frac{\hbar \omega_{\alpha }}{2}\left({\mathrm{p̂}}_{\alpha }^{2}+{\mathrm{q̂}}_{\alpha }^{2}\right)Û=\frac{\hbar \omega_{\alpha }}{2}\left[{\mathrm{q̂}}_{\alpha }^{2}+{\left({\mathrm{p̂}}_{\alpha }+g_{\alpha }\varepsilon_{\alpha }\cdot \mathrm{R̂}\right)}^{2}\right]$$
26

while *V̂̂*_Coul+ext_ remains invariant. Therefore,

$${Ĥ}_{L}\equiv {Û}^{†}{Ĥ}_{v}Û=\overset{N_{e}}{\underset{i=1}{\sum }}\frac{{\mathrm{p̂}}_{i}^{2}}{2m_{i}}+{\mathrm{V̂}}_{\mathrm{Coul}+\mathrm{ext}}+\underset{\alpha }{\sum }\frac{\hbar \omega_{\alpha }}{2}\left[{\mathrm{q̂}}_{\alpha }^{2}+{\left({\mathrm{p̂}}_{\alpha }+g_{\alpha }\varepsilon_{\alpha }\cdot \mathrm{R̂}\right)}^{2}\right]$$
27

Equation 27 is the length-gauge Hamiltonian in the photonic coordinate representation. Expanding the square makes the structure transparent:

$${Ĥ}_{L}=\underset{i}{\sum }\frac{{\mathrm{p̂}}_{i}^{2}}{2m_{i}}+{\mathrm{V̂}}_{\mathrm{Coul}+\mathrm{ext}}+\underset{\alpha }{\sum }\frac{\hbar \omega_{\alpha }}{2}\left({\mathrm{q̂}}_{\alpha }^{2}+{\mathrm{p̂}}_{\alpha }^{2}\right)-\underset{\alpha }{\sum }\mathrm{D̂}\cdot {Ê}_{\alpha }+\underset{\alpha }{\sum }\frac{1}{2\varepsilon_{0}V}{\left(\varepsilon_{\alpha }\cdot \mathrm{D̂}\right)}^{2}$$
28

where the interaction is linear in the field (the −**D̂**·**Ê** coupling), and the last term is the dipole self-energy (*e* ^2^/(2ε_0_ *V*)). Here, ${Ê}_{\alpha }\propto i\sqrt{\hbar \omega_{\alpha }/\left(\varepsilon_{0}V\right)}\varepsilon_{\alpha }\left({â}_{\alpha }-{â}_{\alpha }^{†}\right)$ is the transverse electric field of mode α.

### Momentum Representation and an Equivalent Form

A phase-space rotation of the photonic variables (*q̂*_*α*_, *p̂*_*α*_) |→ (*p̂*_*α*_, −*q̂*_*α*_) (equivalently, *â*_*α*_ |→ *iâ*_*α*_) yields an equally valid representation:

$${Ĥ}_{L}=\underset{i}{\sum }\frac{{\mathrm{p̂}}_{i}^{2}}{2m_{i}}+{\mathrm{V̂}}_{\mathrm{Coul}+\mathrm{ext}}+\underset{\alpha }{\sum }\frac{\hbar \omega_{\alpha }}{2}\left[{\mathrm{p̂}}_{\alpha }^{2}+{\left({\mathrm{q̂}}_{\alpha }-g_{\alpha }\varepsilon_{\alpha }\cdot \mathrm{R̂}\right)}^{2}\right]$$
29

eqs 27 and 29 are related by a canonical 90° rotation in the photon phase space, or equivalently by a global phase of the ladder operators; they are completely equivalent and appear in different papers as distinct length-gauge formulas.

### Diamagnetic Term and Dipole Self-Energy

In the velocity gauge, expanding the kinetic energy produces the diamagnetic term

$${Ĥ}_{\mathrm{dia}}=\underset{i}{\sum }\frac{{q_{i}}^{2}}{2m_{i}}{Â}^{2}$$
30

often called the “seagull” term. It couples two photons to matter in a single vertex and is essential for gauge invariance, correct sum rules, and boundedness of the Hamiltonian. Under the PZW transformation this term maps to the dipole self-energy in ([Disp-formula eq28]),

$$\underset{\alpha }{\sum }\frac{1}{2\varepsilon_{0}V}{\left(\varepsilon_{\alpha }\cdot \mathrm{D̂}\right)}^{2}$$
31

which plays the same stabilizing and gauge-enforcing role in the length gauge. Two-photon processes that are first-order via **Â** ^2^ in the velocity gauge appear at second order in the linear −**D̂**·**Ê** coupling in the length gauge; the total physical predictions coincide.

### Differences of the Two Gauges in Practice

The two Hamiltonians are related by the exact unitary *Ĥ*_L_ = *Û* ^†^ *Ĥ*_V_ *Û* on the full light–matter Hilbert space, hence, they have identical spectra and give identical results for all observables. Differences in which bare Fock-manifolds couple (e.g., Δ*n* = ±1 vs Δ*n* = 0, ±1, ±2) are representation-dependent statements about the bare photon basis, not physical differences. The ladder operators that diagonalize the free field after the PZW transformation are “dressed” by matter, e.g.,

$${\mathrm{b̂}}_{\alpha }\equiv {Û}^{†}{â}_{\alpha }Û={â}_{\alpha }+i\frac{g_{\alpha }}{\sqrt{2}}\varepsilon_{\alpha }\cdot \mathrm{R̂}$$
32

so a “photon” in one gauge is a different superposition of light and matter in the other.

Exact equivalence requires working in the full Hilbert space and keeping **Â** ^2^ (velocity gauge) or the dipole self-energy (length gauge). If one truncates the matter or photon subspaces (few-level or few-photon models), naive truncations can break gauge equivalence. Further practical differences between the two approaches arise from how differential operators are discretized and the selection of basis parameters (e.g., grid spacing in real space representation).

## Notes

Tables S1–S3 contain the molecular geometries used in the paper. The grid parameters and additional computational details are also listed (PDF)
