# Supplementary material for: Quantum-Electrodynamical Time-Dependent Density Functional Theory Description of Molecules in Optical Cavities
Source: J Chem Theory Comput. 2026 Feb 15;22(5):2267–81. doi: 10.1021/acs.jctc.5c01973 (PMC12980728; doi:10.1021/acs.jctc.5c01973)
Supplement: Supplementary file 1 [file ct5c01973_si_001.pdf]

Supporting Information: Quantum-electrodynamical time-dependent density functional theory description of molecules in optical cavities

Yetmgeta Aklilu[1], Matthew Shepherd[2], Cody L. Covington[3], and Kalman Varga[1]

[1] Department of Physics and Astronomy, Vanderbilt University, Nashville, Tennessee 37235, United States
[2]Department of Physics and Astronomy, Auburn University, Auburn, Alabama 36830, United States
[3]Department of Chemistry, Austin Peay State University, Clarksville, Tennessee 37044, United States

*Email:kalman.varga@vanderbilt.edu

This Supporting Information presents the complete molecular geometries and all computational details for the calculations reported in the main text.

LiH

For the LiH system, we positioned the Li and H atoms at a distance of R atomic units from each other along the x-axis. The computational grid was set up with a spacing of 0.2 atomic units and grid dimensions of $91 \times 81 \times 81$. An $N_F = 4$ Fock space was used for the calculations.

BH_3

Table S1: Molecular geometry in atomic units

x	y	z	Atom
0.000000000000	0.000000000000	−0.000000014895	B
0.000000000000	0.000000000000	1.193230299466	H
0.000000000000	1.033367824431	−0.596615068378	H
0.000000000000	−1.033367824431	−0.596615068378	H

The atomic coordinates of the system are provided in the table. The calculations employed a real-space grid with 0.3 a.u. spacing and 71 points along each dimension. The photon Fock space was truncated at $N_F = 2$.

$(H_2)_2$

H_2 dimer setup Each H_2 molecule is aligned along the x-axis with H–H distance of 1.4 a.u., centered at $z = \pm R/2$. The atomic coordinates are $(\pm 0.7, 0, \pm R/2)$, giving an intermolecular separation of R along z. The real-space grid has uniform spacing $\Delta = 0.3$ a.u. and dimensions $60 \times 60 \times 120$. The Fock space is truncated at $N_F = 2$.

Ar_2

For Ar_2, we used the same setup as the H_2 dimer, with the two Ar atoms separated by distance R.

Water dimer

x (a.u.)	y (a.u.)	z (a.u.)	Atom
0.00000000	0.00000000	0.00000000	O
-0.74343740	1.66923350	0.00000000	H
1.82730920	0.00000000	0.00000000	H
0.00000000	0.10079803	-0.21554224	O
0.29870910	-0.68336302	1.40769530	H
0.35982290	-0.98288472	-1.64215370	H

Table S2: First configuration: Coordinates of the water dimer with oxygen atoms at the origin.

x (a.u.)	y (a.u.)	z (a.u.)	Atom
0.00000000	0.00000000	0.00000000	O
-0.74343700	1.66923350	0.00000000	H
1.82730900	0.00000000	0.00000000	H
0.00000000	0.19870480	0.00001281	O
0.29870900	0.11369085	1.56088420	H
0.35982300	0.97133595	-1.92327040	H

Table S3: Second configurations. Coordinates of the water dimer with oxygen atoms at the origin.

The tables show the two water dimer configurations used in the calculations. The water molecules were placed at distance $\pm R$ along the x-axis. The grid spacing is $\Delta = 0.3$ a.u. with dimensions $170 \times 61 \times 61$ and $N_F = 2$.

Water dimer

The HF dimer consists of two HF molecules aligned along the x-axis, each with an H–F bond length of 1.73 a.u. The molecules are positioned symmetrically along the z-axis at $z = \pm R/2$, where R is the intermolecular separation. Specifically, the atomic coordinates are $(\pm 0.8664, 0, \pm R/2)$, with hydrogen at positive x and fluorine at negative x for each molecule. The distance R was systematically varied to obtain the interaction energy as a function of separation. The real-space grid has uniform spacing $\Delta = 0.3$ with dimensions $61 \times 61 \times 120$, extended along the z-direction to accommodate the molecular separation.
